# Cultural Experience Influences Multisensory Emotion Perception in Bilinguals

**DOI:** 10.3390/languages7010012

**Published:** 2022-01-10

**Authors:** Peiyao Chen, Ashley Chung-Fat-Yim, Viorica Marian

**Affiliations:** 1 Department of Psychology, Swarthmore College, Swarthmore, PA 19081, USA; 2 Department of Communication Sciences and Disorders, Northwestern University, Evanston, IL 60208, USA

**Keywords:** emotion, modality interference, cultural immersion, cultural exposure, bilingualism

## Abstract

Emotion perception frequently involves the integration of visual and auditory information. During multisensory emotion perception, the attention devoted to each modality can be measured by calculating the difference between trials in which the facial expression and speech input exhibit the same emotion (congruent) and trials in which the facial expression and speech input exhibit different emotions (incongruent) to determine the modality that has the strongest influence. Previous cross-cultural studies have found that individuals from Western cultures are more distracted by information in the visual modality (i.e., visual interference), whereas individuals from Eastern cultures are more distracted by information in the auditory modality (i.e., auditory interference). These results suggest that culture shapes modality interference in multisensory emotion perception. It is unclear, however, how emotion perception is influenced by cultural immersion and exposure due to migration to a new country with distinct social norms. In the present study, we investigated how the amount of daily exposure to a new culture and the length of immersion impact multisensory emotion perception in Chinese-English bilinguals who moved from China to the United States. In an emotion recognition task, participants viewed facial expressions and heard emotional but meaningless speech either from their previous Eastern culture (i.e., Asian face-Mandarin speech) or from their new Western culture (i.e., Caucasian face-English speech) and were asked to identify the emotion from either the face or voice, while ignoring the other modality. Analyses of daily cultural exposure revealed that bilinguals with low daily exposure to the U.S. culture experienced greater interference from the auditory modality, whereas bilinguals with high daily exposure to the U.S. culture experienced greater interference from the visual modality. These results demonstrate that everyday exposure to new cultural norms increases the likelihood of showing a modality interference pattern that is more common in the new culture. Analyses of immersion duration revealed that bilinguals who spent more time in the United States were equally distracted by faces and voices, whereas bilinguals who spent less time in the United States experienced greater visual interference when evaluating emotional information from the West, possibly due to over-compensation when evaluating emotional information from the less familiar culture. These findings suggest that the amount of daily exposure to a new culture and length of cultural immersion influence multisensory emotion perception in bilingual immigrants. While increased daily exposure to the new culture aids with the adaptation to new cultural norms, increased length of cultural immersion leads to similar patterns in modality interference between the old and new cultures. We conclude that cultural experience shapes the way we perceive and evaluate the emotions of others.

## 1. Cultural Experience Influences Multisensory Emotion Perception in Bilinguals

Every year, thousands of people move to a new country for educational purposes, career opportunities, or personal endeavors. Depending on the destination country, these individuals are exposed to new languages, cultures, and social norms, all of which have been shown to shape the way they perceive, process, and organize information in their environment (Marian, forthcoming). Especially for those trying to adapt to a new culture, the ability to read the socio-emotional cues of others may ease the adjustment process by increasing the likelihood of building new support systems. Although past research has examined the role of cultural immersion on cognitive processes, such as color perception (Athanasopoulos et al. 2010), face perception (Derntl et al. 2009, 2012), object perception (Kitayama et al. 2003), and categorization strategy (Cook et al. 2006), few studies have examined the effect of cultural immersion experience on multisensory emotion perception. The current experiment investigated whether daily exposure to a new culture and the length of immersion influence multisensory emotion perception in bilinguals.

Previous studies have reported that multisensory emotion perception is shaped by the perceiver’s cultural background (Liu et al. 2015a, 2015b; Tanaka et al. 2010). Specifically, individuals from East Asian cultures are more influenced by the emotional expression in the auditory modality (i.e., tone of voice), while individuals from Western cultures are more influenced by the emotional expression in the visual modality (i.e., facial expressions). These cultural differences in modality interference have been attributed to display rules (Liu et al. 2015a, 2015b; Tanaka et al. 2010), which are a set of rules learned from an early age through socialization that regulate how to appropriately express emotions (Ekman and Friesen 1971). In East Asian collectivist societies, individuals tend to suppress and control their emotions and personal preferences to accommodate the thoughts and feelings of others (Markus and Kitayama 1991; Matsumoto et al. 1998; Morling et al. 2002; Weisz et al. 1984). On the contrary, in Western individualistic societies, emotions are expressed with the intention to influence others, leading to more direct eye contact amongst Westerners than Easterners (Argyle et al. 1986; McCarthy et al. 2006, 2008). An open question is whether the degree of interference from the visual or auditory modality for individuals who migrate to a new country is fixed across cultures (i.e., they maintain their old pre-existing schemas) or changes depending on the cultural context.

Although there is a prescribed set of rules within each culture, culturally-specific schemas and behaviors are not static and can change as a consequence of new cultural experiences. For example, after being immersed in the United Kingdom for 3.5 years, immigrants from Greece began to perceive the colors blue and green in a way that resembled native speakers from the United Kingdom (Athanasopoulos et al. 2010). Of particular relevance to the current study, Liu et al. (2017) found that Chinese immigrants who moved to Canada were more distracted by irrelevant facial expressions, mirroring the behavior of North American participants. However, Chinese immigrants’ brain activity revealed that they were equally distracted by the irrelevant facial and vocal expressions, mirroring the brain activity of native Mandarin speakers from China (Liu et al. 2015a). These results suggest that cultural immersion leads to culturally-specific changes in emotion perception, at least at the behavioral level. Note that the participants in the study by Liu et al. (2017) migrated to Canada between the ages of 10 and 18 and were tested exclusively on emotional stimuli from their old Eastern culture (i.e., Asian face with Mandarin speech). Therefore, some of the participants had more experience immersed in the North American culture than in Eastern cultures; it remains unknown how they would perceive multisensory emotions in their new culture (i.e., Caucasian face with English speech). We aim to build on these findings by having participants judge multisensory emotional stimuli from their old and new culture and splitting participants into two groups based on the duration of time they lived in the United States. This allows us to investigate whether the length of immersion influences multisensory emotion perception.

The process by which emotional behaviors change due to repeated interactions with a new cultural context has been described as emotional acculturation (Cosedine et al. 2014; De Leersnyder et al. 2013). De Leersnyder et al. (2011) compared the emotional patterns of first-generation Korean immigrants living in the United States and Turkish first-generation immigrants living in Belgium to the emotional patterns of members of the majority culture in each country. Participants rated the extent to which they experienced feeling angry, ashamed, happy, proud, and respectful on a scale from 1 (Not at all) to 7 (Extremely) in response to a set of scenarios. The emotional responses of first-generation immigrants were lower than the average responses of members of the majority culture. However, with increased exposure to the new culture and repeated daily social interactions with members of the majority culture, the emotional patterns of first-generation minorities became more similar to those of the majority culture. These findings from the emotional acculturation literature suggest that immigrants may maintain the emotional patterns from their heritage culture, but increased exposure to the new culture changes their emotional patterns to align with the new culture.

While increased cultural experience can result in new patterns of emotion processing, it does not necessarily mean that the behavioral patterns that were developed in the old cultural context are completely discarded. In fact, those who identify with two cultures sometimes exhibit different behavioral patterns, depending on the cultural context (Berry 1997). This is known as the Cultural Frame Switching hypothesis (Hong et al. 2000; LaFromboise et al. 1993), and refers to bicultural individuals accessing and shifting their mental schemas based on the culture that was most recently activated. Culturally-specific images have also been found to elicit different cultural tendencies in attribution styles (Benet-Martínez et al. 2002; Hong et al. 2003; Hong et al. 2000), personality traits (Morris and Mok 2011; Ramírez-Esparza et al. 2006), and self-concept or identity (Cheng et al. 2006; Ross et al. 2002; West et al. 2018). Language has also been shown to elicit culture-specific behaviors (De Leersnyder et al. 2020; Marian and Kaushanskaya 2004; Panayiotou 2004; Perunovic et al. 2007). For example, Greek-English bilinguals reacted to the same story differently depending on the language in which the story was read (Panayiotou 2004). When the story was read to them in Greek, the bilinguals reported feeling concerned for the protagonist. In contrast, when the story was read to them in English, the bilinguals reported feeling indifferent towards the protagonist. Cultural cues can influence social cognition and potentially play a unique role in shaping the way bilinguals who migrate to a new country evaluate multisensory emotions.

In previous studies examining the Cultural Frame Switching hypothesis, the cultural context was primed with cultural images or by languages that were independent from the task itself. For multisensory emotion processing, the cultural context is primed with stimuli that are embedded within the task (i.e., language and face). For bilinguals who learn to communicate in a new language and recognize the faces of a new racial group, both the visual input (i.e., facial expressions of different racial groups) and the auditory input (i.e., vocal expressions in different languages) may serve as strong cues to signal different cultural contexts.

The present study examined whether cultural experience affects multisensory emotion perception when a bilingual moves from an East Asian culture to a Western culture. Chinese-English bilinguals who were born and raised in China and moved to the United States were presented with face-language pairs from their old Eastern culture (i.e., Asian face with Mandarin speech) and new Western culture (i.e., Caucasian face with English speech). There are three possible ways that bilinguals migrating from China to the United States would exhibit modality interference. The first possibility is that bilinguals would adopt new Western cultural norms by increasing their reliance on the visual modality when presented with Eastern and Western emotional information, replicating the behavioral findings by Liu et al. (2017). The second possibility is that bilinguals would hold both Eastern and Western norms and exhibit culturally-specific behaviors depending on the cultural context, supporting the Cultural Frame Switching hypothesis (Hong et al. 2000; LaFromboise et al. 1993). Specifically, they would show greater interference from the visual modality when presented with emotional input from the West, and greater interference from the auditory modality or equivalent interference from both modalities when presented with emotional input from the East. The third possibility is that bilinguals would maintain their old Eastern cultural norms by showing greater interference from the auditory modality across both cultures, in line with the electrophysiological findings by Liu et al. (2017).

In addition, we investigated whether the amount of daily exposure to a new culture and the length of immersion influence multisensory emotion perception. Both the amount of daily exposure and the length of immersion have been previously found to influence emotional acculturation (Cosedine et al. 2014; De Leersnyder et al. 2011). Specifically, we compared Chinese-English bilinguals with shorter immersion experience to those with longer immersion experience in the U.S., as well as Chinese-English bilinguals with low daily exposure to those with high daily exposure to the U.S. culture. A closer look at these two factors would enable us to tease apart the role of accumulated experience (i.e., immersion) from that of everyday experience (i.e., exposure).

## 2. Materials and Methods

### 2.1. Participants

Forty-nine Chinese-English bilinguals between the ages of 18 and 35 were recruited through posters around campus and university email listservs. Participants were compensated for their participation with an Amazon gift card at the rate of $10/hour. Chinese-English bilinguals were recruited based on four inclusionary criteria: (1) being proficient in English and Mandarin, (2) living in North America at the time of testing, (3) being born and raised in China to Chinese parents, and (4) previously living in China for at least 6 years before moving to a Western country. The study was conducted remotely via the Internet and informed consent was obtained by all participants in their native language.

Three participants did not complete the task and another four participants performed at or below chance level on the emotion recognition task. In addition, one participant had recently moved to the United States, which resulted in a mean length of time in China that was 3 standard deviations greater than the group’s mean. The remaining 41 participants (31 females, *M*_*Age*_ = 24.07 years, *SD*_Age_ = 3.53) had spent an average of 19.3 years in China (*SD* = 3.93 years, range: 9 years to 26 years) and, by the time of testing, had spent an average of 4.70 years in Western countries (*SD* = 3.51 years, range: 4 months to 14 years). Participants’ language background information was obtained using an adapted version of the Language Experience and Proficiency Questionnaire (Marian et al. 2007). All participants acquired Mandarin from birth and rated their proficiency in Mandarin as 9.53 out of 10 (*SD* = 0.63). On average, participants learned English before the age of 7 (*M* = 6.95, *SD* = 3.37) and rated their proficiency in English as 7.54 out of 10 (*SD* = 1.21). Their daily exposure to Eastern and Western cultures was 46.54% (*SD* = 17.04) and 49.02% (*SD* = 19.04), respectively. There was no relation between immersion length and percentage of daily exposure to Western culture, *r*(41) = 0.12, *p* = 0.45. All participants had normal or corrected-to-normal vision and no hearing impairments. The study was approved by the local Institutional Review Board.

### 2.2. Materials

#### Language Experience and Proficiency Questionnaire (LEAP-Q)

An adapted version of the LEAP-Q (Marian et al. 2007) was used to assess each participant’s linguistic and cultural backgrounds. The first set of questions contained demographic questions, including age, years of formal education, gender, and any history of hearing or vision impairments. The second set of questions targeted their language history. Participants were asked to report the languages they spoke, including any non-native languages. For each language listed, participants rated their level of proficiency from 1 to 10 (1 = very low and 10 = perfect) and reported the age of acquisition. The third set of questions pertained to the participant’s cultural background. Participants were asked to list the cultures they identified with. For each culture listed, participants rated the extent to which they identified with each culture on a scale from 0 to 10 (0 = no identification and 10 = complete identification), and the percentage of time spent exposed to each culture. Lastly, participants listed the countries they had previously lived in, and the duration of time spent in each country.

### 2.3. Stimuli

#### 2.3.1. Vocal Stimuli

Twenty Mandarin and 20 English pseudo-sentences were selected from two validated vocal emotional stimuli databases (Mandarin: Liu and Pell 2012; English: Pell et al. 2009). Pseudo-sentences followed the segmental properties of each language respectively but included no semantic information. The pseudo-sentences were spoken in five basic emotions (happiness, sadness, disgust, fear, and anger) by 2 female and 2 male native speakers of each language, resulting in a unimodal voice list of 20 pseudo-sentences (4 voices × 5 emotions = 20 pseudo-sentences) in each language. Based on the normed data within each database, the Mandarin and English pseudo-sentences were matched on recognition rate, emotional intensity, and duration, *t*s < 1 (Table 1).

#### 2.3.2. Face Stimuli

Twenty Asian faces and 20 Caucasian faces were selected from two databases (Asian faces: Taiwanese Facial Expression Image Database by Chen and Yen (2007); Caucasian faces: Karolinska Directed Emotional Faces Database by Lundqvist et al. (1998)). Five different facial expressions (happiness, sadness, disgust, fear, and anger) were displayed by 2 female and 2 male actors from each database, resulting in a unimodal face list of 20 faces for each culture (4 faces × 5 emotions = 20 faces). Based on the normed data in each database, the Asian and Caucasian faces were matched on recognition rate and emotional intensity, *t*s < 1 (Table 1). To maintain consistency in size, brightness, and contrast, the images were re-processed in GIMP 2.9.8 (GIMP Development Team 2015) to the same dimension (345 pixels wide × 430 pixels high), resolution (300 dpi), and intensity (grayscale).

#### 2.3.3. Bimodal Face-Voice Stimuli

**Figure 1.**
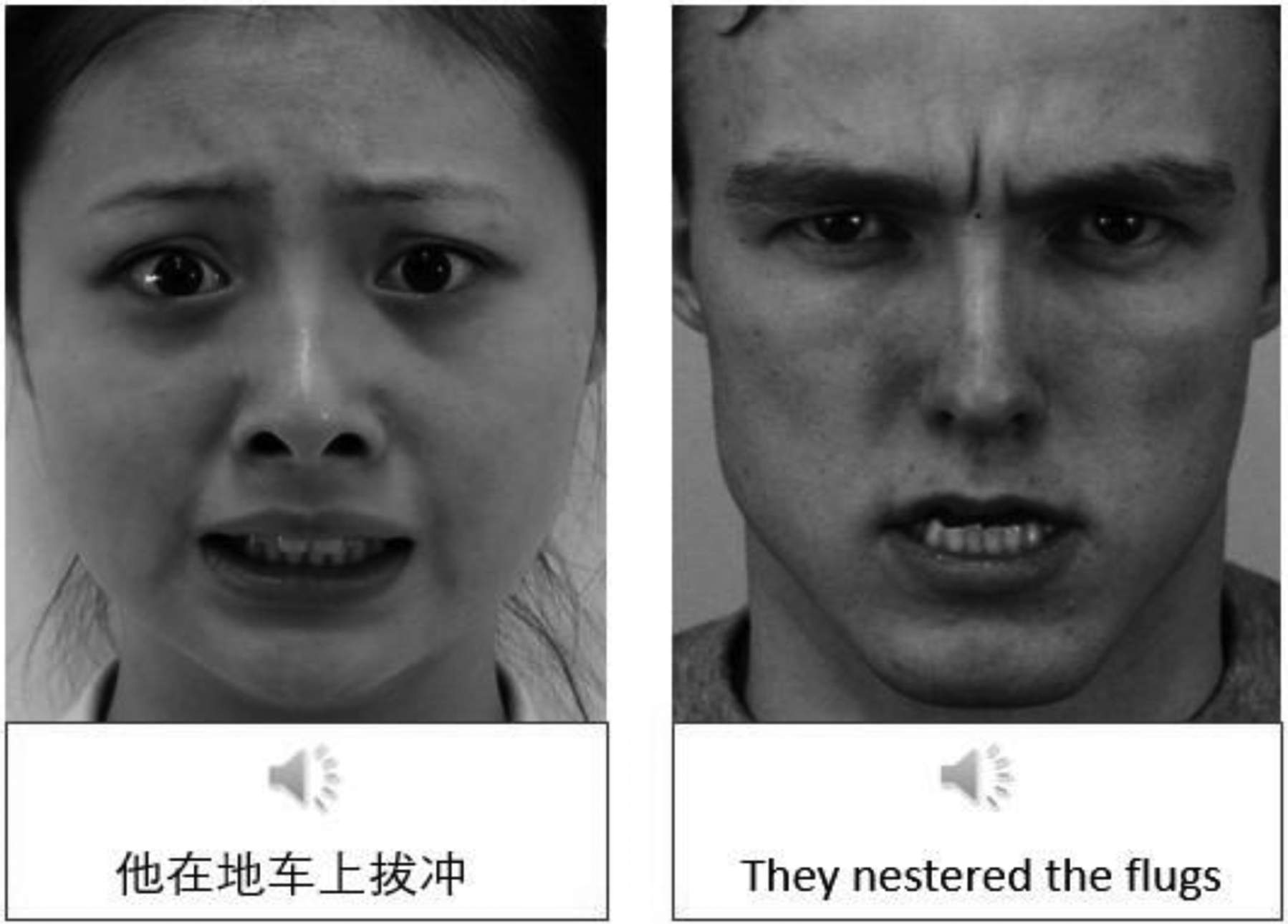
An example of bimodal stimuli from each culture. In the left-hand panel, an Asian face is paired with meaningless Mandarin speech (East Asian culture). In the right-hand panel, a Caucasian face is paired with meaningless English speech (Western culture). (Asian face—Image ID fea118: Adapted with permission from Chen and Yen (2007); Mandarin pseudo-sentence: Adapted with permission from Liu and Pell (2012); Caucasian face—Image ID AM08ANS: Adapted with permission from Lundqvist et al. (1998); English pseudo-sentence: Adapted with permission from Pell et al. (2009).)

For each culture, bimodal face-voice stimuli were created by pairing a unique voice with a unique face of the same gender. The same voice was always paired with the same face to maintain consistency in identity (Figure 1). The emotion displayed across modalities could either be the same (congruent condition; e.g., happy face and happy voice) or different (incongruent condition; e.g., happy face and sad voice). Because a total of five emotions were used in the study with four different speakers/actors, each face was paired once with the voice of the same emotion for a total of 20 bimodal congruent trials and once with each of the remaining four emotions for a total of 80 bimodal incongruent trials. As a result, four bimodal lists were created, each containing 20 congruent trials and 20 of the 80 incongruent trials.

#### 2.3.4. Fillers

To discourage participants from developing a strategy, 12 bimodal filler trials with new faces and voices were used. Three trials contained a red dot (radius = 20 mm in size) on the cheek of the face and another three trials had a 500 ms beep inserted in the speech stream.

### 2.4. Design and Procedure

The emotion recognition task consisted of two tasks. In the face task, participants were instructed to identify the emotion of the face and ignore the emotion of the voice. In the voice task, participants were instructed to identify the emotion of the voice and ignore the emotion of the face. Each participant was assigned one of the four bimodal lists, a unimodal face list, and a unimodal voice list from each culture. The same bimodal list was used for the face task and voice task.

For each trial, a prompt instructing participants to identify either the emotion of the voice (i.e., voice task; “Judge the Voice Emotion”) or the emotion on the face (i.e., face task; “Judge the Face Emotion”) appeared first. Once the participant clicked on the prompt, a bimodal or a unimodal stimulus appeared in the middle of the screen. On unimodal face trials, a face appeared for a random duration between 1500 to 2000 ms with a 100 ms interval, which is consistent with the duration of 95% of the vocal stimuli. On unimodal voice trials, a fixation cross appeared in the middle of the screen while a pseudo-sentence was presented through the participant’s speakers. On bimodal face-voice trials, a face appeared for the duration of the speech. For all three trial types, participants were then presented with a display of five emotion words in English (happiness, sadness, disgust, fear, and anger) and instructed to select the emotion they perceived by clicking the box next to the emotion as quickly as possible. After selecting an emotion, they rated the intensity of the perceived emotion on a scale from 0 (not intense at all) to 6 (extremely intense). On filler trials, a beep in the speech stream or a red dot on the cheek of the face appeared for 500 ms within the last 600 ms to 700 ms of a trial. Instead of rating the intensity of the emotion, participants reported whether they saw a red dot on the face or heard a beep by clicking “Yes” or “No”.

The face and voice tasks were presented in separate blocks, with the order of presentation counterbalanced across participants. Within each task, unimodal, bimodal congruent, bimodal incongruent, and filler trials from both cultures were intermixed and randomly presented. The face and voice tasks did not differ in the number of switches between cultures, *t*(44) = −0.51, *p* = 0.61, and between trial types, *t*(44) = −0.99, *p* = 0.33. In addition, for the bimodal trials, the number of switches between congruent and incongruent trials was equivalent in the face and voice tasks, *t*(44) = −0.41, *p* = 0.68. Participants were provided 10 practice trials at the start of each task and three breaks that were embedded throughout. The entire testing session was 60 to 90 minutes long.

## 3. Results

Response times (RTs) were measured from the onset of the display with the five emotions until a response was made. Only correct trials were included in the analyses for RTs and intensity ratings. RTs below 500 ms and above 5000 ms were discarded from the analyses. For accuracy rates and intensity ratings, modality interference was computed as the difference between the bimodal congruent trials and bimodal incongruent trials. For RTs, modality interference was computed as the difference between the bimodal incongruent trials and bimodal congruent trials. The higher the modality interference in the face task, the greater the interference from the irrelevant voice. The higher the modality interference in the voice task, the greater the interference from the irrelevant face.

### 3.1. Modality Interference across Participants

**Figure 2.**
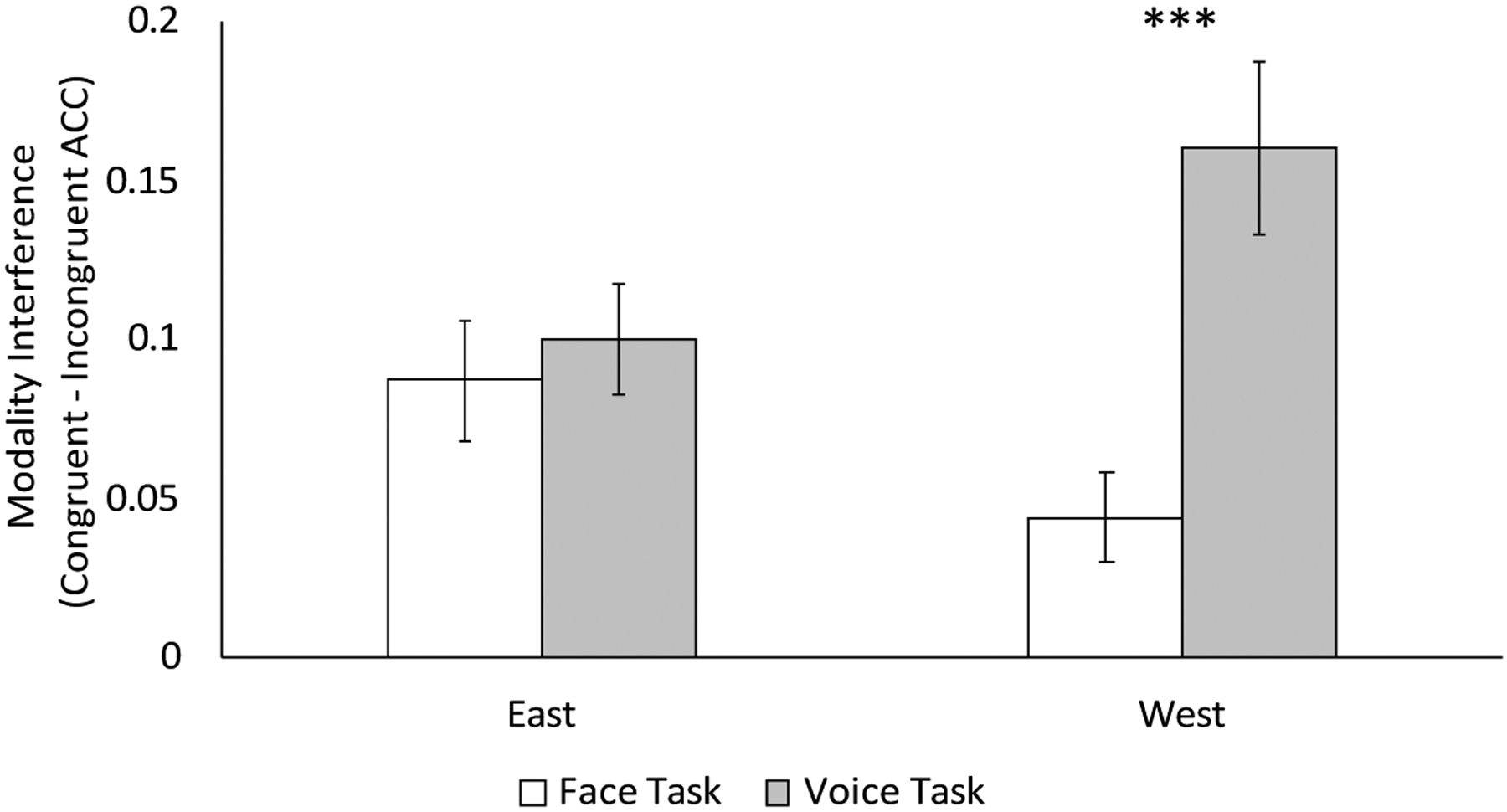
Modality interference effects in accuracy rates (bimodal congruent trials minus bimodal incongruent trials). A larger modality interference in the face task represents greater interference from the auditory modality, whereas a larger modality interference in the voice task represents greater interference from the visual modality. Chinese-English bilinguals experienced a larger modality interference in the voice task than in the face task for the emotional information from the West (i.e., visual interference). Error bars represent standard error. *** *p* < 0.001.

The mean accuracy rates, response times, and intensity ratings by condition, culture, and task are presented in Table 2. A 2-way ANOVA with task (face vs. voice) and culture (East vs. West) as within-subjects factors on accuracy rates yielded a main effect of task, *F*(1,40) = 10.72, *p* = 0.002, *η*_*p*_^2^ = 0.21, showing that modality interference was larger in the voice task (*M* = 0.13, *SE* = 0.019) than the face task (*M* = 0.065, *SE* = 0.012), but no effect of culture was observed, *F* < 1. The task by culture interaction was significant, *F*(1,40) = 9.13, *p* = 0.004, *η*_*p*_^2^ = 0.19. When the emotional input was from the West, there was a larger modality interference in the voice task than the face task, *F*(1,40) = 15.52, *p* < 0.001, *η*_*p*_^2^ = 0.28, suggesting that participants experienced greater interference from the visual modality than auditory modality. No difference between modalities was found for emotional input from the East, *F* < 1 (Figure 2). To examine the Cultural Frame Switching hypothesis, we also examined the interaction of task and culture by comparing Eastern and Western stimuli for the voice and face tasks separately. There was a larger modality interference for Western stimuli than Eastern stimuli on the voice task, *F*(1,40) = 7.73, *p* = 0.008, *η*_*p*_^2^ = 0.16, suggesting that participants experienced greater interference from the irrelevant face when evaluating emotional speech from the West. The modality interference was larger for Eastern stimuli than for Western stimuli on the face task, but this difference did not reach significance *F*(1,40) = 3.59, *p* = 0.065, *η*_*p*_^2^ = 0.082, suggesting that participants showed a tendency towards greater interference from the irrelevant speech when evaluating facial expressions from the East. The analyses on RTs and intensity ratings yielded no significant effects or interactions, all *ps* > 0.16.

### 3.2. Long vs. Short Immersion

To examine the effect of immersion length, participants were divided into two groups using a median split based on the duration of time spent in the United States. Bilinguals in the shorter immersion group (*N* = 22) spent less than or equal to 4 years in the United States, whereas those in the longer immersion group (*N* = 19) spent more than 4.5 years in the United States. The groups were matched on Chinese proficiency, age of English acquisition, and years of formal education, *p*s > 0.06. The bilinguals in the short immersion group were younger, *t*(39) = −2.19, *p* = 0.034, and rated their English proficiency lower than the bilinguals in the long immersion group, *t*(39) = −2.11, *p* = 0.041 (Table 3). The difference in English proficiency is not surprising given that the bilinguals in the long immersion group had lived in an English-speaking country for a longer period of time and were likely more confident in their English-speaking abilities. English proficiency correlated with the accuracy rates on the voice task when listening to English speech, *r*(41) = −0.32, *p* = 0.044. Age and years of formal education did not correlate with any of the dependent measures on the emotion recognition task (*p*s > 0.12). Separate three-way ANOVAs with task (face vs. voice) and culture (East vs. West) as the within-subjects factors and length of immersion (short vs. long) as the between-subjects factor were performed on accuracy rates, RTs, and intensity ratings. The mean accuracy rates, RTs, and intensity ratings by condition, task, culture, and length of immersion are presented in Table 4.

**Figure 3.**
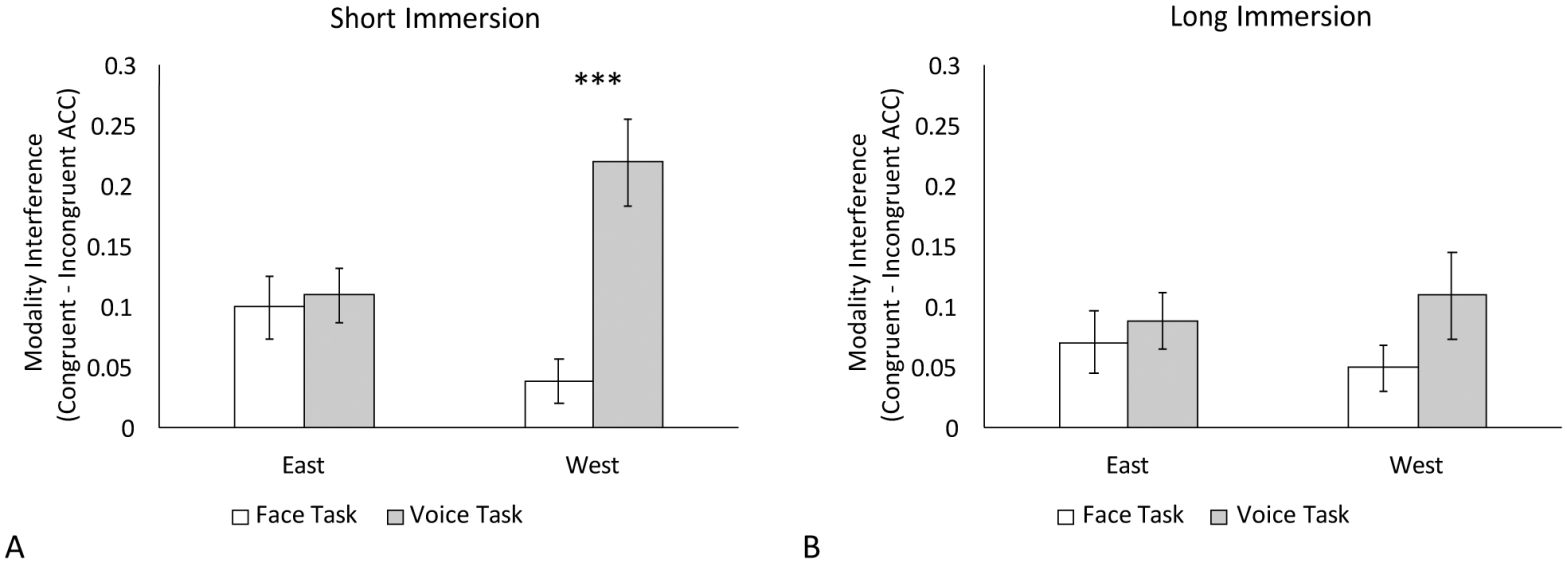
Modality interference effects in accuracy rates (bimodal congruent trials minus bimodal incongruent trials) for Chinese-English bilinguals immersed in North America for a short duration (**A**) and long duration (**B**). The short immersion group showed a larger modality interference in the voice task than the face task for the emotional information from the West (i.e., visual interference). The long immersion group showed no differences between tasks in both cultures. Error bars represent standard error. *** *p* < 0.001.

In the accuracy rates analyses, there was a marginally significant three-way interaction between task, culture, and immersion, *F*(1,39) = 3.63, *p* = 0.064, *η*_*p*_^2^ = 0.085. The main effect of task, *F*(1,39) = 10.25, *p* = 0.003, *η*_*p*_^2^ = 0.21, and the interaction between task and culture, *F*(1,39) = 8.83, *p* = 0.004, *η*_*p*_^2^ = 0.19, were significant, but the main effect of immersion was not, *F*(1,39) = 2.22, *p* = 0.14. To breakdown the three-way interaction, separate two-way ANOVAs were conducted for each immersion group. Among the Chinese-English bilinguals who lived in the U.S. for a shorter duration, the task by culture interaction was significant, *F*(1,21) = 16.59, *p* < 0.001, *η*_*p*_^2^ = 0.44. Modality interference was larger in the voice task than the face task when the emotional input was from the West, *F*(1,21) = 16.71, *p* < 0.001, *η*_*p*_^2^ = 0.44, but there were no differences in modality interference between the two tasks when the emotional input was from the East, *F* < 1 (Figure 3). Furthermore, the main effect of task was significant, *F*(1,21) = 8.46, *p* = 0.008, *η*_*p*_^2^ = 0.29, in which the voice task (*M* = 0.16, *SE* = 0.029) produced a larger modality interference than the face task (*M* = 0.069, *SE* = 0.016). The effect of culture was not significant, *F* < 1. Among the Chinese-English bilinguals who were immersed in the U.S. for a longer duration, neither the main effects nor the interaction were significant, *p*s > 0.13. These findings demonstrate that with increased immersion experience, bilinguals experience similar patterns in modality interference across their two cultures. Analyses of RTs and intensity ratings yielded no significant main effects or interactions, *p*s > 0.15.

### 3.3. Low vs. High Daily Exposure

To examine the effect of cultural exposure on multisensory emotion perception, participants were divided into low (less than 50%; *N* = 18) and high (50% or more; *N* = 23) levels of cultural exposure to Western culture using a median split, based on their self-reported percentage of daily exposure to Western culture. The low and high daily exposure groups were matched on age, years of formal education, English proficiency, Chinese proficiency, and age of English acquisition, all *p*s > 0.082 (Table 3). Separate three-way ANOVAs with task (face, voice) and culture (East, West) as the within-subjects factors and exposure (low vs. high) as the between-subjects factor were performed on accuracy rates, RTs, and intensity ratings. Mean accuracy rates, RTs, and intensity ratings by condition, task, culture, and amount of exposure to the U.S. culture are shown in Table 5.

**Figure 4.**
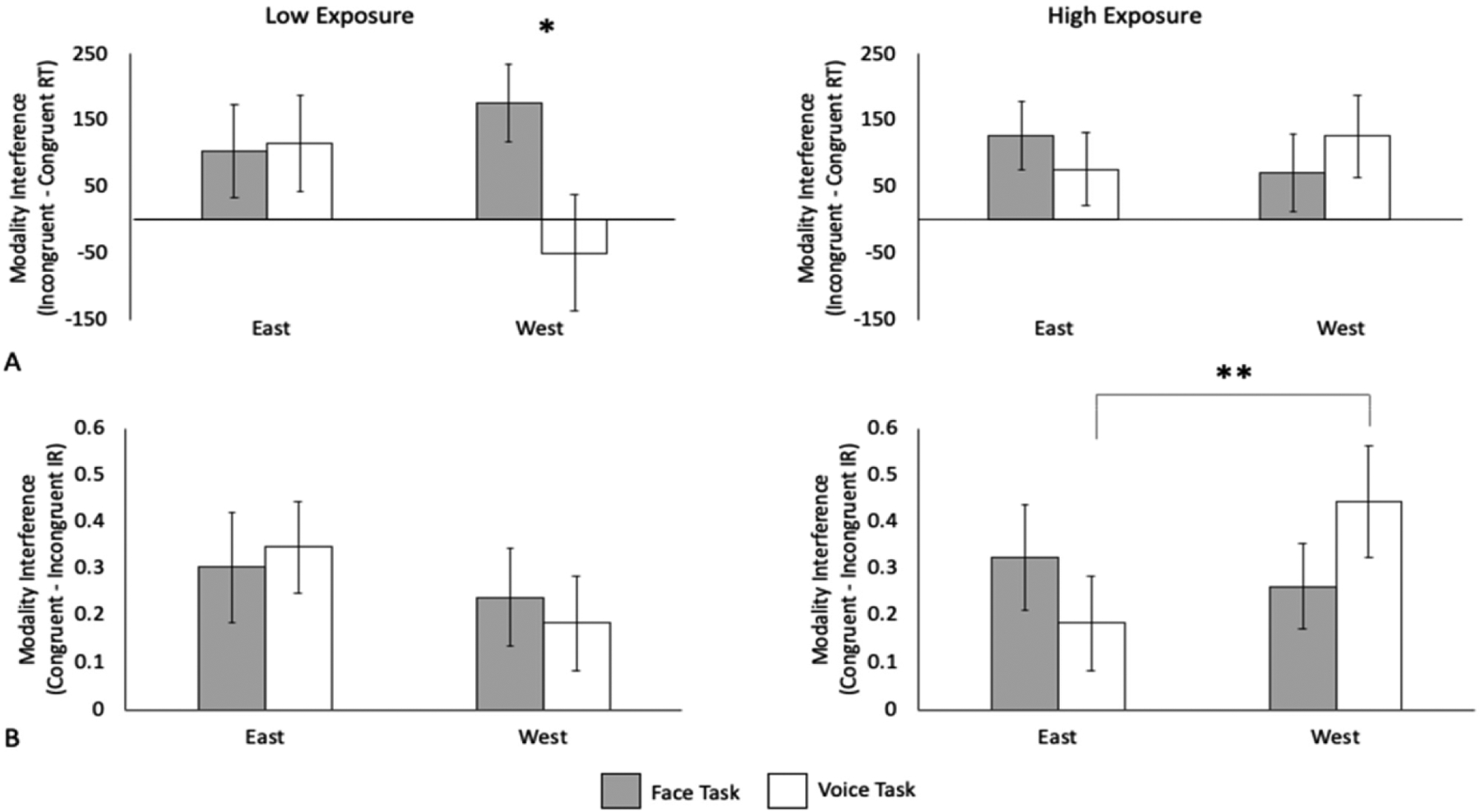
Modality interference effects on (**A**) response times and (**B**) intensity ratings for bilinguals with low (left panels) and high (right panels) levels of exposure to the U.S. culture. The low exposure group showed a larger modality interference in the face task than in the voice task for emotional information from the West (i.e., auditory interference). The high exposure group showed a larger modality interference for Western than Eastern emotional information in the voice task, but no differences in modality interference between cultures in the face task. * *p* < 0.05, ** *p* < 0.01.

In the RT analyses, the three-way interaction of task, culture, and exposure was marginally significant, *F*(1,39) = 4.03, *p* = 0.052, *η*_*p*_^2^ = 0.094. Separate two-way ANOVAs were performed for each exposure group. In the bilingual group with low exposure to the U.S. culture, there was a marginally significant culture by task interaction, *F*(1,17) = 3.96, *p* = 0.063, *η*_*p*_^2^ = 0.19. A larger modality interference was found in the face task than the voice task when the emotional input was from the West, *F*(1,17) = 6.59, *p* = 0.020, *η*_*p*_^2^ = 0.28, but there were no differences between tasks when the emotional input was from the East, *F* < 1 (Figure 4a). These findings suggest that Chinese-English bilinguals with low exposure to the U.S. culture are more impacted by vocal expressions than facial expressions when presented with emotional stimuli from the West. This pattern of auditory interference coincides with their East Asian culture. The main effects of task and culture were not significant, *p*s > 0.092. In the bilingual group with high exposure to the U.S. culture, none of the main effects and interactions reached significance, *Fs* < 1.

In the intensity ratings analyses, the culture by exposure interaction was significant, *F*(1,39) = 4.45, *p* = 0.041, *η*_*p*_^2^ = 0.10, as was the three-way interaction between task, culture, and exposure, *F*(1,39) = 5.10, *p* = 0.030, *η*_*p*_^2^ = 0.12 (Figure 4b). To breakdown the interaction, separate two-way ANOVAs were conducted for each exposure group. In the bilingual group with low exposure to the U.S. culture, the main effects and interactions were not significant, *p*s > 0.24. In the bilingual group with high exposure to the U.S. culture, the culture by task interaction was significant, *F*(1,22) = 5.82, *p* = 0.025, *η*_*p*_^2^ = 0.21. Specifically, there was a larger modality interference in the voice task when the emotional stimuli were from the West (*M* = 0.44, *SE* = 0.12) than the East (*M* = 0.18, *SE* = 0.10), *F*(1,22) = 9.41, *p* = 0.006, *η*_*p*_^2^ = 0.30, but no differences in modality interference between cultures were found in the face task, *F* < 1.

In the accuracy rates analyses, there was a main effect of task, *F*(1,39) = 10.38, *p* = 0.003, *η*_*p*_^2^ = 0.21, with the voice task (*M* = 0.13, *SE* = 0.020) producing a larger modality interference than the face task (*M* = 0.065, *SE* = 0.012). The task by culture interaction was also significant, *F*(1,39) = 8.65, *p* = 0.005, *η*_*p*_^2^ = 0.18. There was a larger modality interference in the voice task (*M* = 0.17, *SE* = 0.028) than the face task (*M* = 0.045, *SE* = 0.014), but only when the stimuli were from the West, *F*(1,39) = 14.88, *p* < 0.001, *η*_*p*_^2^ = 0.28, and not the East, *F* < 1. No other effects were significant, *F*s < 1.

## 4. Discussion

The current study examined whether cultural experience, including length of immersion and amount of daily exposure to a new culture, affects multisensory emotion perception. Overall, bilinguals who migrated to the United States from China experienced greater interference from the visual modality than the auditory modality when asked to judge emotional stimuli from the West. However, when asked to judge emotional stimuli from the East, the bilinguals in the current study experienced similar degrees of interference from both modalities. Interestingly, these patterns were observed in the Chinese-English bilinguals who have been immersed in the American culture for a short duration of time. The differences across modalities and between cultures disappeared in bilinguals with longer immersion experience. Bilinguals with low exposure to the U.S. culture displayed auditory interference, whereas bilinguals with high exposure to the U.S. culture displayed visual interference when presented with Western emotional information. These results reveal that cultural immersion and exposure play important, but distinct, roles in multisensory emotion perception.

The bilinguals in the current study were more impacted by the facial cues than vocal cues when evaluating Western emotional information, replicating previously-observed visual interference patterns exhibited by Westerners (Liu et al. 2015b; Tanaka et al. 2010). When evaluating Eastern emotional information, the bilinguals were equally distracted by the auditory and visual modalities, replicating Liu et al.’s behavioral findings (2015b). At first glance, these findings appear to support the Cultural Frame Switching (CFS) hypothesis. However, this overall modality interference pattern is more similar to that of the bilinguals with shorter immersion experience than that of the bilinguals with longer immersion experience, making the CFS interpretation less plausible. In our findings, a noticeable pattern was that bilinguals with shorter immersion experience relied heavily on the visual modality when presented with emotional information from the West. As immersion experience increased, modality interference across cultures appeared to converge, suggesting that bilinguals with longer immersion experience may merge the two cultures into one. If the CFS hypothesis was supported, bilinguals with longer immersion would have shown visual interference when perceiving emotional information from the West.

Furthermore, a closer look at Figure 3 reveals that the difference between bilinguals with shorter and longer immersion was in the condition where participants were asked to judge English speech, *t*(39) = 2.10, *p* = 0.042. There was a positive correlation between length of immersion in the U.S. and accuracy in judging English speech, showing that accuracy increased as the length of immersion increased, *r*(41) = 0.31, *p* = 0.046. This positive correlation suggests that greater interference from Caucasian faces when judging the English speech could be due to limited English familiarity in bilinguals with shorter immersion experience. This limited familiarity in the auditory modality might cause the bilinguals to over-compensate by excessively relying on the visual modality. In fact, previous studies have shown that during multisensory emotion processing, individuals tend to rely more on the modality they are most familiar with (Chen 2019; Collignon et al. 2008). Therefore, it is likely that the differences in modality interference between Eastern and Western cultures among bilinguals with shorter immersion experience was driven by familiarity rather than switching between different cultural contexts. Future research is needed to disentangle the relative contribution of familiarity from the length of immersion on multisensory emotion perception in individuals who migrate to a new country and are in the process of adopting new cultural norms.

In contrast to the findings by Liu et al. (2017), we did not observe visual interference in the condition where participants were presented with Asian facial expressions and Mandarin speech. The discrepancy between our findings and theirs may be due to the sample under investigation. The participants in Liu et al. (2017) had spent relatively equal time in Eastern and Western countries, whereas the participants in the current study spent more time in an Eastern country than a Western country. The participants in our study may have had less experience with Western cultures than the participants in Liu et al. (2017). Another possibility is that the participants in Liu et al.’s study became less familiar with Mandarin, as they were using English for 68–81 hours per week on average. Previous work has shown that perceivers increase their reliance on the visual modality when multisensory emotional input is less familiar (Chen 2019). Therefore, it is possible that the bilinguals in Liu et al.’s study may have increased their reliance on the visual modality (i.e., Asian face) because the auditory input was less familiar.

Compared with length of immersion, daily exposure to the new culture had a different effect on emotion perception. Response times revealed that bilinguals with low daily exposure to the U.S. culture relied more on the auditory modality when evaluating Western emotional information, but did not rely more on the auditory modality when evaluating Eastern emotional information. A possible explanation of this puzzling finding is that participants consistently relied on the more-familiar modality when evaluating unfamiliar emotional input from the West. However, when evaluating familiar emotional input from the East, modality reliance may have varied depending on ties to the old culture, willingness to assimilate into the new culture, and duration of immersion, with potentially opposite direction of effects across participants. For example, participants who feel strongly about preserving their Eastern culture may be more likely to continue relying on the auditory modality, whereas participants seeking to integrate into the new culture may be less likely to rely on the auditory modality. The opposite patterns may be cancelling each other out, resulting in no overall modality interference for Eastern emotional information in the low exposure participants.

For the individuals with high daily exposure to the U.S. culture, the pattern of intensity ratings showed that they might be adjusting their reliance on the visual modality depending on the context of the interaction, which is consistent with the Cultural Frame Switching hypothesis (Hong et al. 2000; LaFromboise et al. 1993). Specifically, we observed that there was increased interference from the visual modality when participants were presented with emotional information from the West compared to when they were presented with information from the East. Therefore, daily exposure to the new culture seems to change our perceptions to reflect new cultural norms, consistent with the findings from the emotional acculturation literature (Cosedine et al. 2014; De Leersnyder et al. 2011).

The differences between the low and high daily exposure groups may be due to each group’s experience with and degree of confidence in recognizing the emotions of Western individuals, as well as to distinctions between what is being assessed by RT versus intensity ratings. On the emotion recognition task, RTs were measured as the amount of time required to select the perceived emotion from five alternatives. The longer RTs in the low exposure group may be indicative of greater uncertainty discriminating between multiple emotions (Arndt et al. 2018; Lischetzke et al. 2011). Intensity ratings, on the other hand, required participants to compare how intense a perceived emotion was relative to input with no emotion and may be capturing a combined cognitive appraisal of the subjective experience (i.e., inferences about the mental state; Matsumoto 1999) and the valence of the emotion (e.g., pleasant or unpleasant; Russell 1980; Sutton et al. 2019). Because response times and intensity measures are potentially tapping into different cognitive phenomena and may have different degrees of sensitivity when measuring modality interference, more research is needed to understand how various measures tap into different aspects of emotional processing.

It remains unclear how much daily exposure is necessary for the Cultural Frame Switching (CFS) to emerge in emotion perception. Does the CFS pattern emerge predominantly amongst those who divide their time more evenly across cultures or do individuals have to immerse themselves completely into the new culture to reduce the effects from the old culture? A possible future direction would be to test participants who grew up with both cultures and have similar levels of exposure to each culture. Another consideration related to testing the CFS hypothesis is to separate the influence of cultural immersion from familiarity. For Chinese-English bilinguals in the current study, the less familiar auditory input (i.e., English) resulted in increased visual interference. However, this increased visual interference is also predicted by having increased Western cultural exposure, making it hard to separate the effect of familiarity from the effect of cultural immersion. An alternative would be to test English speakers moving from a Western culture to China. According to the familiarity account, the less familiar input (i.e., Mandarin speech) would lead to increased visual interference in the new Eastern culture. The CFS hypothesis, on the other hand, would predict an increased auditory interference when processing emotional input from the East.

The current study has several limitations that will need to be addressed in future research. First, the linguistic environment may have impacted the Cultural Frame Switching hypothesis. The task instructions and language of testing were in English, which may have increased Western-culture-specific behaviors. Second, the absence of a monolingual and monocultural control group limits the interpretation of results. In an attempt to address this limitation in future research, we are currently developing a study that will focus on emotion recognition in monolingual native English speakers in the United States and monolingual native Mandarin speakers in China (it is difficult to ascertain the monocultural identity of even monolingual participants, however, because people can identify with multiple cultures despite living in the same country or speaking one language; indeed, the definition of culture is open to debate). Lastly, the face stimuli used in the current study were static images of faces rather than dynamic facial expressions. Moving faces have been shown to improve facial and emotional recognition and lead to more intense responses than static faces (see Alves 2013; Xiao et al. 2014). In addition, contextual information, such as the visual scene, description of the event, and body language, can also serve as important cues for detecting emotions (Aviezer et al. 2017; Barrett et al. 2011). Future research should consider using audio and video recordings to study emotion processing of dynamic facial expressions in variable contexts.

In conclusion, immersion experience and daily exposure to a new culture impacted multisensory emotion perception in different ways. As length of immersion increased, Chinese-English bilinguals demonstrated a similar pattern in modality interference across cultures. This finding suggests that bilinguals who move from an Eastern to a Western culture might merge the norms from their two cultures and demonstrate similar modality interference when perceiving emotional input from both cultures. Furthermore, an individual’s exposure to the cultural and social norms of the new culture predicts the likelihood of exhibiting a modality interference pattern that is more common in the new culture. We conclude that length of immersion in a culture and amount of cultural exposure interact to shape perceptual and cognitive processes, such as emotion perception.

## Figures and Tables

**Table 1.** Recognition accuracy and emotional intensity ratings for the sentences and faces from each culture.

Stimuli	Culture	Percent Recognition Rate	Emotional Intensity (0 to 5 for Sentences and 0 to 9 for Faces)	Duration (in Seconds)
Sentences	Mandarin	86 (7.3)	3.3 (0.6)	1.78 (0.26)
	English	88 (7.4)	3.4 (0.4)	1.79 (0.19)
Faces	Asian	83 (12.1)	5.6 (0.6)	-
	Caucasian	84 (12.6)	5.7 (1.0)	-

**Table 2.** Mean accuracy rates (ACC), response times in ms (RT), and intensity ratings (IR) across cultures, tasks, and conditions (standard deviations are in parentheses).

Measure	Culture	Task	Bimodal Congruent	Bimodal Incongruent	Modality Interference
ACC	East	Face	0.86 (0.097)	0.77 (0.14)	0.087 (0.12)
		Voice	0.88 (0.11)	0.78 (0.15)	0.10 (0.11)
	West	Face	0.80 (0.095)	0.75 (0.11)	0.044 (0.090)
		Voice	0.78 (0.12)	0.61 (0.16)	0.16 (0.18)
RT	East	Face	1484 (310)	1601 (317)	117 (268)
		Voice	1583 (370)	1676 (343)	93 (281)
	West	Face	1474 (290)	1591 (334)	117 (269)
		Voice	1757 (416)	1806 (377)	49 (338)
IR	East	Face	4.05 (0.90)	3.74 (0.94)	0.31 (0.51)
		Voice	4.19 (0.77)	3.93 (0.93)	0.25 (0.46)
	West	Face	3.96 (0.91)	3.71 (0.95)	0.25 (0.43)
		Voice	3.96 (0.94)	3.63 (0.92)	0.33 (0.52)

**Table 3.** Language background of short vs. long immersion and low vs. high exposure groups.

	Short Immersion	Long Immersion	*p*-Value	Low Exposure	High Exposure	*p*-Value
N	22	19		18	23	
Gender	3 M, 19 F	6 M, 13 F		4 M, 14 F	5 M, 15 F	
Age in Years	23.00 (2.74)	25.32 (3.99)	0.034 *	23.39 (3.87)	24.61 (3.23)	0.28
Years of Education	16.52 (2.54)	18.23 (3.19)	0.06	16.58 (2.70)	17.91 (3.07)	0.16
Chinese Proficiency	9.68 (0.48)	9.37 (0.76)	0.12	9.56 (0.62)	9.52 (0.67)	0.87
English Proficiency	7.18 (1.10)	7.95 (1.22)	0.041 *	7.17 (1.15)	7.83 (1.19)	0.08
English AoA	6.86 (2.13)	7.06 (4.48)	0.86	7.47 (2.42)	6.55 (3.96)	0.40

Note.

* *p* < 0.05.

AoA = Age of Acquisition. Chinese proficiency and English proficiency were rated out of 10.

**Table 4.** Mean accuracy rates (ACC), response times in ms (RT), and intensity ratings (IR) across cultures, tasks, length of immersion, and conditions (standard deviations are in parentheses).

Culture	Measure	Task	Immersion	Bimodal Congruent	Bimodal Incongruent	Modality Interference
East	ACC	Face	Short	0.84 (0.093)	0.74 (0.10)	0.10 (0.13)
			Long	0.88 (0.099)	0.81 (0.16)	0.071 (0.11)
		Voice	Short	0.87 (0.098)	0.77 (0.17)	0.11 (0.12)
			Long	0.89 (0.11)	0.80 (0.12)	0.090 (0.084)
	RT	Face	Short	1521 (318)	1688 (341)	167 (282)
			Long	1441 (304)	1501 (259)	60 (245)
		Voice	Short	1661 (437)	1791 (347)	130 (300)
			Long	1492 (256)	1543 (294)	51 (258)
	IR	Face	Short	4.13 (0.89)	3.83 (0.92)	0.29 (0.48)
			Long	3.97 (0.92)	3.63 (0.97)	0.34 (0.56)
		Voice	Short	4.26 (0.73)	3.92 (0.98)	0.33 (0.44)
			Long	4.11 (0.82)	3.94 (0.88)	0.16 (0.46)
West	ACC	Face	Short	0.77 (0.092)	0.73 (0.084)	0.039 (0.083)
			Long	0.83 (0.090)	0.78 (0.12)	0.050 (0.10)
		Voice	Short	0.78 (0.097)	0.57 (0.15)	0.22 (0.18)
			Long	0.77 (0.14)	0.66 (0.16)	0.11 (0.16)
	RT	Face	Short	1495 (300)	1637 (347)	141 (261)
			Long	1448 (283)	1538 (319)	89 (284)
		Voice	Short	1875 (460)	1940 (405)	65 (378)
			Long	1620 (316)	1650 (277)	30 (294)
	IR	Face	Short	4.08 (0.86)	3.86 (.85)	0.22 (0.32)
			Long	3.82 (0.97)	3.53 (1.04)	0.28 (0.54)
		Voice	Short	4.08 (0.84)	3.72 (0.97)	0.36 (0.60)
			Long	3.82 (1.04)	3.52 (0.86)	0.30 (0.43)

**Table 5.** Mean accuracy rates (ACC), response times in ms (RT), and intensity ratings (IR) across cultures, tasks, level of exposure to Western culture, and conditions (standard deviations are in parentheses).

Culture	Measure	Task	Exposure	Bimodal Congruent	Bimodal Incongruent	Modality Interference
East	ACC	Face	Low	0.85 (0.098)	0.77 (0.16)	0.078 (0.11)
			High	0.87 (0.096)	0.78 (0.12)	0.094 (0.13)
		Voice	Low	0.89 (0.097)	0.79 (0.16)	0.097 (0.10)
			High	0.88 (0.11)	0.78 (0.15)	0.10 (0.11)
	RT	Face	Low	1491 (371)	1596 (373)	104 (298)
			High	1478 (262)	1606 (273)	128 (248)
		Voice	Low	1686 (459)	1801 (406)	115 (308)
			High	1502 (266)	1578 (253)	76 (264)
	IR	Face	Low	4.00 (1.03)	3.71 (1.06)	0.30 (0.50)
			High	4.09 (0.80)	3.76 (0.86)	0.32 (0.54)
		Voice	Low	4.07 (0.76)	3.72 (0.93)	0.35 (0.41)
			High	4.28 (0.78)	4.10 (0.91)	0.18 (0.48)
West	ACC	Face	Low	0.76 (0.10)	0.71 (0.13)	0.050 (0.11)
			High	0.83 (0.080)	0.79 (0.077)	0.039 (0.075)
		Voice	Low	0.77 (0.12)	0.60 (0.15)	0.17 (0.19)
			High	0.78 (0.12)	0.62 (0.17)	0.16 (0.17)
	RT	Face	Low	1451 (298)	1628 (337)	177 (246)
			High	1491 (289)	1562 (336)	71 (283)
		Voice	Low	1927 (494)	1877 (421)	−50 (369)
			High	1624 (289)	1750 (338)	126 (298)
	IR	Face	Low	3.99 (0.97)	3.75 (1.02)	0.24 (0.45)
			High	3.94 (0.88)	3.68 (0.91)	0.26 (0.43)
		Voice	Low	3.78 (0.93)	3.60 (0.85)	0.18 (0.42)
			High	4.09 (0.94)	3.65 (0.99)	0.44 (0.57)

## Data Availability

The data presented in this study are available upon request from the corresponding author. The data are not publicly available because public sharing of the data was not approved by the Institutional Review Board and because participants did not provide permission to have their data shared publicly at the time of testing.
